# A strong association of axillary osmidrosis with the wet earwax type determined by genotyping of the *ABCC11 *gene

**DOI:** 10.1186/1471-2156-10-42

**Published:** 2009-08-04

**Authors:** Motoi Nakano, Nobutomo Miwa, Akiyoshi Hirano, Koh-ichiro Yoshiura, Norio Niikawa

**Affiliations:** 1 Department of Reconstruction and Plastic Surgery, Nagasaki University Graduate School of Biomedical Sciences, Nagasaki, Japan; 2 Department of Human Genetics, Nagasaki University Graduate School of Biomedical Sciences, Nagasaki, Japan; 3 Research Institute of Personalized Health Sciences, Health Sciences University of Hokkaido, Hokkaido, Japan; 4 Solution Oriented Research of Science and Technology (SORST), Japan Science and Technology Agency (JST), Tokyo, Japan

## Abstract

**Background:**

Two types of cerumen occur in humans: the wet type with brownish, sticky earwax, and the dry type with a lack of or reduced ceruminous secretion. The wet type is common in populations of European and African origin, while the dry type is frequently seen in Eastern Asian populations. An association between axillary odor and the wet-type earwax was first identified approximately 70 years ago. The data were based on a phenotypical analysis of the two phenotypes among the Japanese by a researcher or by self-declaration of the subjects examined, and were not obtained using definite diagnostic methods. Recently, we identified a single-nucleotide polymorphism (SNP; rs17822931) of the *ABCC11 *gene as the determinant of the earwax types. In the present study, to determine whether the SNP can serve as a diagnostic marker for axillary osmidrosis (AO), we examined genotypes at rs17822931 in 79 Japanese AO individuals. AO was defined here as a clinical condition of individuals with a deep anxiety regarding axillary odor and had undergone the removal of bilateral axillary apocrine glands.

**Results:**

A comparison of the frequencies of genotypes at rs17822931 in the 79 AO individuals and in 161 Japanese from the general population showed that AO was strongly associated with the wet earwax genotype. A total of 78 (98.7%) of 79 AO patients had either the GG or GA genotype, while these genotypes were observed in 35.4% (57/161) of the subjects from the general population (*p *< 1.1 × 10^-24^, by Fisher's exact test).

**Conclusion:**

The strong association between the wet-earwax associated *ABCC11-*genotypes (GG and GA) and AO identified in this study indicates that the genotypes are good markers for the diagnosis of AO. In addition, these results suggest that having the allele G is a prerequisite for the axillary odor expression. In other words, the ABCC11 protein may play a role in the excretory function of the axillary apocrine gland. Together, these results suggest that when an AO individual visiting a hospital is diagnosed with dry-type earwax by *ABCC11*-genotyping, surgical removal of their axillary glands may not be indicated.

## Background

Apocrine and/or eccrine glands in the human body cause odor, especially from the axillary and pubic apocrine glands. As in other mammals, the odor may have a pheromone-like effect on the opposite sex. Although the odor does not affect health, axillary osmidrosis (AO) is a condition in which an individual feels uncomfortable with their axillary odor, regardless of its strength, and may visit a hospital. Surgery to remove the axillary gland may be performed on demand. AO is likely an oligogenic trait with rs17822931 accounting for most of the phenotypic variation and other unidentified functional variants accounting for the remainder. However, no definite diagnostic criteria or objective measuring methods have been developed to characterize the odor, and whether an individual suffers from AO depends mainly on their assessment and/or on examiner's judgment. Human body odor may result from the breakdown of precursors into a pungent odorant by skin bacteria [1], but it is unclear if AO is this type of odor.

The consistency of human earwax is a dimorphic genetic trait and two distinct types are known: the wet type characterized as sticky, brownish earwax, and the dry type characterized as scurf or scales of the external ear canal. The wet type is completely dominant to the dry type, and is very common in populations of European and African origin (~95% and ~100%, respectively) [2-7]. In contrast, the dry type is frequently seen in Eastern Asian populations, with the prevalence of the wet earwax being ~15% in Japan, ~5% in Korea and ~10% among the Han Chinese [2,7]. We have recently identified an earwax determining SNP, c.538G>A (rs17822931), in the *ABCC11 *gene [6], and confirmed the gene as an earwax-type determinant. We calculated the allele frequencies in various ethnic populations [6], which can now be retrieved from existing databases. From the HapMap data, the G-allele (defining the wet type) frequency is estimated to be 1.000 in the Yoruba population (Africa), 0.875 in CEPH families (Europe), and 0.111 in Tokyo habitants (Japan), and the frequencies estimated from ALFRED (the allele frequency database) show overall accordance with those from the HapMap data.

A relationship between axillary odor and the wet-type earwax was first noticed among the Japanese population concurrent with the first discovery of the earwax type as a Mendelian trait. Japanese clinicians assert an association between axillary odor and earwax type; however, since no definite diagnostic criteria or measuring methods were available for the two traits, the data is based on observations of the two respective traits.

Here we report the result of a genotyping study examining rs17822931 of the *ABCC11 *gene in Japanese individuals with AO, and discuss the ABCC11 genotype as a diagnostic tool for AO.

## Results

We analyzed a total of 79 AO individuals from either Nagasaki or Okinawa prefectures. Of the 79 AO patients, 5 were GG homozygotes, 73 were GA heterozygotes, and 1 was an AA homozygote. Therefore, 98.7% (78/79) of the AO individuals had the GG or GA genotype (Table 1). In contrast, the GG and GA genotypes were observed in 35.4% (57/161) of the overall population in the prefectures. In Nagasaki, GG, GA and AA genotypes are observed in 2, 39 and none of the 41 AO individuals, and in 4, 36 and 87 of the general population samples, respectively. Likewise in Okinawa prefecture, GG, GA and AA genotypes comprised 3, 34, and 1 of the AO patients, and 2, 15, and 17 of the general Okinawans population sample. Fisher's exact test showed a strong association between the wet type genotype and AO (*p *< 8.4 × 10^-17 ^for the Nagasaki habitants, and *p *< 3.0 × 10^-6 ^for the Okinawans) (Table 1). Although G allele frequency is considerably higher among Okinawans than habitants in other Kyushu areas [6], no significant difference was detected between general Nagasaki and Okinawa populations (*p *> 0.06). This may be due to the relatively small number of samples from Okinawa in this study. Fisher's exact test of the combined data showed a strong association of the wet type genotype and AO (*p *< 1.1 × 10^-24^).

**Table 1:** Association of AO with the wet earwax type

Subject studied	Genotype at the rs17822931 locus (earwax phenotype)					
	
	GG	GA	(wet type)	AA	(dry type)	total
Individuals with AO (Kyushu)	5	73	(78)^a^	1	(1)^a^	79
General habitants in Kyushu	6	51	(57)^a^	104	(104)^a^	161

total			(135)^a^		(105)^a^	240

Individuals with AO (Okinawa)	3	34	(37)^b^	1	(1)^b^	38
General habitants in Okinawa	2	15	(17)^b^	17	(17)^b^	34

total			(54)^b^		(18)^b^	72

Individuals with AO (Nagasai)	2	39	(41)^c^	0	(0)^c^	41
General habitants in Nagasaki	4	36	(40)^c^	87	(87)^c^	127

total			(81)^c^		(87)^c^	168

^a^*p *< 1.1 × 10^-24^, ^b^*p *< 3.0 × 10^-6^, ^c^*p *< 8.4 × 10^-17^: all comparisons were performed under a dominant model. All statistical analyses were done by Fisher's exact test. Hardy-Weinberg equilibrium was supported under the observed allele frequency in control samples by Exact Hardy-Weinberg test (*p*-value > 0.1) [18]. These comparisons were performed using plink software [19].

## Discussion

We have shown that AO in the Japanese population is strongly associated with the wet earwax genotypes, with the results supporting the 70-year-old data of a strong, positive association between the two traits. If all AO in the Japanese is a Mendelian trait and is primarily determined by allele G at the c.538G/A polymorphic site in the *ABCC11 *gene, an all-or-none result would have been expected. In other words, under this condition, AA homozygotes should not have been included in the AO group, and all individuals with GG or GA genotype should have AO. However, a single individual with the AA genotype was present in our series of AO samples, and, thus, not all the samples showed deterministic association (Table 1). Since no objective way to quantify or qualify axillary odor is available and the diagnosis of any given AO individual is made on the basis of their history and complaints, we focused in this study only on AO individuals who visited plastic surgery clinics and did not assess the odor quantity of GG and GA individuals in the general population. Some individuals without AO may exist, and their axillary odor may be controlled by other genes and/or factors that modify the *ABCC11 *function. Primary (cause unknown and possibly genetic) and secondary (multiple causes including anxiety, menopause, hyperthyroidism, stroke, drugs, amongst other causes) hyperhidrosis may be an explicable factor for AO in individuals with the AA genotype. It is plausible that the *ABCC11 *gene primarily determines the quality of AO, while modifiers play a role in its quantity, such as pre-determination regarding the number of the apocrine glands in the axilla.

The *ABCC11 *gene, which encodes MRP8, is expressed in various types of tissues [8,9] and is a member of the ATP-binding cassette transporter gene family [10]. Most ABC transporter proteins are localized to the plasma membrane and are ATP-dependent transporters of a broad range of compounds [11], such as cyclic nucleotides, lipophilic anions (glutathione-conjugated LTC4), sulfated steroids (DHEAS and E_1_3S), glucuronides (E_2_17βG), bile constituents (glycocholate and taurocholate), and monoglutamates (methotrexate) [12]. MRP8 is localized to the apical membrane of MDCK cells when expressed artificially [13]. Since most MRP proteins transport substrates from the inside to the outside of the cell, certain compound(s) that may cause axillary odor are secreted through MRP8 in the axillary apocrine gland. The axillary gland of individuals with the wet earwax type may secrete the materials more highly than that of the dry type individuals, as seen in a previous in vitro experiment [6].

The nature of axillary odor and whether the axillary odorants come directly from secreted materials of the axillary gland are unknown. Some carboxylic acids were reported to be possible components of such odorants. Zeng et al. [14] demonstrated that (*E*)-3-methylhex-2-enoic acid (3M2H) is a key odorant component, and its hydrated analogue (*RS*)-3-hydroxy-3-methylhexanonoic acid (HMHA) was the most abundant pungent odorant in the axilla [15]. Sweat itself does not smell, but skin bacteria (*Corynebacteria*) transform non-odoriferous precursors in sweat into a pungent odorant [1]. In addition, a specific Zn-dependent *Nα*-acyl-glutamine aminoacylase (N-AGA) in the bacteria has been reported to catalyze a reaction that produces 3M2H and HMHA from *Nα*-acyl-glutamine conjugates secreted into sweat in the axilla [15]. Natsch et al. [16] claimed that since there are other odoriferous materials, the proportion of these components causes odor variance among individuals. However, since axillary odor can be detected immediately after sweating (especially a large amount of rapid nervous sweating), there is insufficient time for bacterial growth. In addition, since the odor does not completely disappear by washing with water, but disappears with the use of soap, axillary odorants may contain certain lipophilic components, as does earwax. Therefore, it remains unclear whether these precursors in sweat are substrates of MRP8 and their secretion might be reduced or lacking in individuals with dry type earwax.

Recently, we examined the biochemical characteristics of the G allele (wild type allele) and A allele (mutant allele) [17]. Our results showed that the wild type ABCC11 protein is glycosylated and localized to the ceruminous gland membrane, but mutant ABCC11 from the A allele is not glycosylated and is degraded rapidly by the proteasome system. Degradation by the proteasome is not a complete process and some of the protein is localized on the cell surface membrane; therefore, mutant ABCC11 may retain some excretion function [17]. In this context, body odorant derived from ABCC11 function may represent a quantitative trait that depends on the protein levels on the cell surface. Previously, we reported on the excretion properties of wild type and mutant ABCC11 using cGMP as a substrate. Since cGMP is not an authentic substrate for odor, further work is needed to identify the odorant in AO or from the ABCC11 substrate not only for biochemical characterization, but also as an objective tool to measure the axillary odor

## Conclusion

In the present study, we demonstrated a strong association between the wet earwax genotype and AO. Our results suggest that genotyping at the rs17822931 locus may be a useful tool for supplementing the diagnosis of patients that present at clinic with OA. A result of this study suggests that the presence of allele G at the rs17822931 locus is a prerequisite for AO. Since almost all of the patients complaining of AO in this study did not have the AA genotype (78/79), we suggest that further study may prove rs17822931 to provide useful additional information in diagnosing AO patients. Two key issues remain to be addressed. The first is that, although the estimated sensitivity of the genotype diagnostic test from this study is high (approximately 99%) for patients who present with AO, the specificity of the test in this context is low, with 35% of controls also carrying the G risk allele. Second, it is vital that further research identify more objective clinical definitions of AO, since the sensitivity and specificity data presented in this study are conditional upon the subjective diagnosis of AO.

## Methods

### Subjects studied

The examinees included 79 Japanese individuals with AO, who were examined at plastic surgery clinics. Of the 79 AO samples, 41 were from Nagasaki prefecture and the remaining 38 were from Okinawa prefecture. Both prefectures are located in the Kyushu area, the most western district of Japan. Wet earwax frequency is different among prefectures of Japan [2], so we divided the samples into two groups based on the prefectures. One hundred twenty seven samples in Nagasaki and 34 samples in Okinawa were used as general population controls for chi-square test. These samples were previously collected for calculating the allele frequency of rs17822931 in ABCC11. All of the samples from AO cases and controls were collected with written informed consent, and protocols for the present study were approved by the Committee for the Ethical Issues on Human Genome and Gene Analysis at Nagasaki University.

In this study, AO individuals were defined as those who were anxious about axillary odor and had received a surgical operation in the clinics to remove their axillary apocrine glands. In general, some Japanese are very sensitive and nervous of body odor and often visit the clinics, probably because the majority of the population have faint odor. From this background, plastic surgeons are familiar with AO and the collection of AO patients is easy. However, no objective diagnostic methods are available for axillary odor. Therefore, diagnosis of AO was made through self-declaration by the individual and through the clinician's judgment at interview prior to the operation. Earwax type was not considered for the AO diagnosis. Although AO due to primary and secondary hyperhidrosis was excluded as much as possible, individuals with such conditions may have been included in our samples. If we collected samples from individuals with faint axillary odor (or without odor), well-trained plastic surgeons who collected the "AO" patient judged the axillary odor. In this situation, our association could be defined as a double blind study, but it is difficult to smell the axilla in the general population. Therefore, we focused our interest on the measurement the sensitivity of the earwax genotype to judge AO. When objective diagnostic methods are available for axillary odor, a complete double blind study will be feasible.

### Genotyping at the SNP site (rs17822931) and association study

Genomic DNA was extracted from all examinees, and was subjected to PCR-based genotyping at the SNP site, rs17822931 (c.538G>A), in the *ABCC11 *gene. For the TaqMan genotyping, VIC-labeled TaqMan MGB wet-probe (5'-CAGTGTACTCGGGCCAG-3') and FAM-labeled TaqMan MGB dry-probe (5'-CAGTGTACTCAGGCCAG-3') were used as hydrolyzing probes, while EW-ampF (5'-CTTCTGGGCATCTGCTTCTG-3') and EW-ampR (5'-CAAACCTCACCAAGTCTGCCA-3') were used as amplification primers. Reactions were carried out using TaqMan Universal PCR Master Mix (AppliedBiosystems). Homozygotes for allele A were categorized to have the dry earwax type and others the wet type [6]. The number of AO individuals within each respective genotype was statistically compared with that in the general population by Fisher's exact test, and Hardy-Weinberg equilibrium was tested under the observed allele frequency [18,19].

## Authors' contributions

MN collected samples from the Nagasaki area and extracted DNA. NM extracted DNA from tissue samples of AO individuals and performed genotyping. AH participated in the design of the study, collected samples from the Okinawa area and analyzed the clinical data of AO patients. KY performed statistical analysis. KY and NN participated in the design of the study and supervised the above researchers/clinicians and prepared the manuscript. All authors read and approved the final manuscript.
